# Phagocytic Glial Cells in Brain Homeostasis

**DOI:** 10.3390/cells10061348

**Published:** 2021-05-29

**Authors:** Rena Kono, Yuji Ikegaya, Ryuta Koyama

**Affiliations:** 1 Laboratory of Chemical Pharmacology, Graduate School of Pharmaceutical Sciences, The University of Tokyo, Bunkyo-ku, Tokyo 113-0033, Japan; rena.k322@gmail.com (R.K.); yuji@ikegaya.jp (Y.I.); 2 Institute for AI and Beyond, The University of Tokyo, Tokyo 113-0033, Japan; 3 Center for Information and Neural Networks, National Institute of Information and Communications Technology, Suita City 565-0871, Japan

**Keywords:** microglia, astrocyte, phagocytosis, lysosome, Aβ

## Abstract

Phagocytosis by glial cells has been shown to play an important role in maintaining brain homeostasis. Microglia are currently considered to be the major phagocytes in the brain parenchyma, and these cells phagocytose a variety of materials, including dead cell debris, abnormally aggregated proteins, and, interestingly, the functional synapses of living neurons. The intracellular signaling mechanisms that regulate microglial phagocytosis have been studied extensively, and several important factors, including molecules known as “find me” signals and “eat me” signals and receptors on microglia that are involved in phagocytosis, have been identified. In addition, recent studies have revealed that astrocytes, which are another major glial cell in the brain parenchyma, also have phagocytic abilities. In this review, we will discuss the roles of microglia and astrocytes in phagocytosis-mediated brain homeostasis, focusing on the characteristics and differences of their phagocytic abilities.

## 1. Introduction

Phagocytosis is a dynamic process that consists of several steps. First, the targets of phagocytosis are recognized by phagocytic receptors or are ingested through nonspecific mechanisms. Next, the phagocytosed targets are encapsulated and incorporated into the early endosome, where they are sorted for the degradation pathway or recycling pathway. In the degradation pathway, early endosomes mature into late endosomes, which fuse with lysosomes and acidic vesicles containing proteases, and finally the phagocytosed targets are degraded.

The targets of phagocytosis include pathogens, cell debris, and abnormally aggregated proteins, and the clearance of these proteins is important not only for the immune system but also for metabolism. In the central nervous system (CNS), microglia are considered to be the major phagocytes. Recently, however, accumulating evidence has suggested that astrocytes are also capable of phagocytosis under both physiological and pathological conditions [1,2]. However, the roles of microglia and astrocytes in phagocytosis and their differences, if any, remain largely unclear. It is sometimes argued that either microglia or astrocytes are the “major” phagocytes in the CNS, but this argument may be misguided. Microglia are mesoderm-derived cells, while astrocytes are ectoderm-derived cells. When two types of glial cells of different origins, which are sometimes referred to as brain immune cells, perform phagocytosis in the brain, which is mainly composed of ectodermal cells, the mechanism by which phagocytic targets are recognized by these two cell types can be different. Thus, we hypothesized that the difference in phagocytic target recognition mechanisms might be the basis for the differences in the roles of microglia and astrocytes in phagocytosis.

Here, we outline the main targets of phagocytosis of both cell types, cell debris, amyloid-β (Aβ), and synapses, while comparing the characteristics and molecular mechanisms of phagocytosis in microglia and astrocytes. For cell debris and Aβ, we will focus on the 3 steps of phagocytosis: recognition by receptors, degradation in lysosomes, and the fate of both phagocytes and the phagocytosed targets after ingestion. For synapses, we will discuss the first steps, recognition by receptors, and the characteristics of the phagocytosed synapses.

## 2. Phagocytosis of Cell Debris

**Figure 1:**
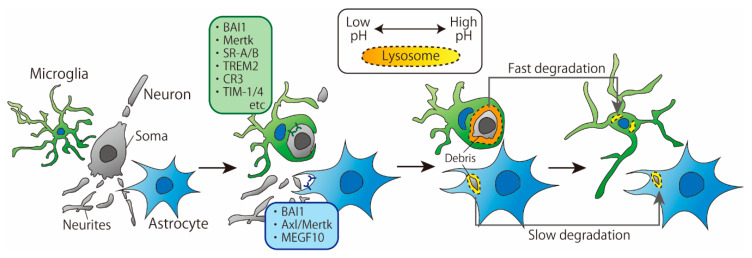
**Time course of phagocytosis of cellular debris by microglia and astrocytes.** Microglia tend to phagocytose larger cell debris than astrocytes. The pH of microglial lysosome is lower than that of astrocytes, which may result in faster degradation of debris.

Recently, a growing number of studies have directly and simultaneously compared phagocytosis by microglia and astrocytes. Damisah et al. observed the phagocytic abilities of microglia and astrocytes around an apoptotic neuron using in vivo two-photon imaging and 2Phatal, a technique that induces apoptosis in a single neuron by irradiating the Hoechst 33342-labeled neuronal nucleus with a laser [3]. The results showed that microglia and astrocytes exhibited spatiotemporally distinct phagocytic patterns: microglia phagocytosed the soma and proximal dendrites of apoptotic neurons, while astrocytes phagocytosed distal fine dendrites. Moreover, in the absence of microglia, astrocytes took a longer time to remove apoptotic neurons than microglia, which is consistent with a previous report [4] (Figure 1). In this section, we discuss the differences in microglia- and astrocyte-mediated phagocytosis of cell debris based on the underlying cellular and molecular mechanisms.

### 2.1. Recognition by Receptors

The first step in phagocytosis is the recognition of cell death signals expressed on apoptotic cells by receptors on phagocytes. A variety of microglial receptors and downstream signaling involved in the phagocytosis of cell debris have already been reported and well summarized [5]. On the other hand, astrocytes also have several receptors that recognize cell debris. Brain-specific angiogenesis inhibitor-1 (BAI1), which can recognize phosphatidylserine (PS), is expressed on astrocytes and is involved in the phagocytosis of apoptotic thymocytes [6,7]. Furthermore, it has been reported in vitro that the loss of multiple epidermal growth factor-like domains 10 (MEGF10) impairs phagocytosis in embryonic fibroblasts and that the ligand of MEGF10 is complement C1q, which is the triggering molecule that activates the classical complement pathway [8]. In addition, when apoptotic neurons were cocultured with healthy neurons, astrocytes and oligodendrocytes, the expression of MEGF10 in cell lysates increased, suggesting that MEGF10 plays an important role in the clearance of cell debris [9]. The coculture system contained three types of cells, and MEGF10 was thought to be expressed mainly by astrocytes.

Konishi et al. showed that microglial debris is engulfed by astrocytes when microglial apoptosis is induced [10]. To examine the molecules involved in astrocyte phagocytosis, the authors used an in vitro phagocytosis assay system in which apoptotic microglia were added to a primary culture of astrocytes. In this system, knockdown of MEGF10 with siMEGF10 in astrocytes did not impair astrocyte-mediated phagocytosis of apoptotic microglia, whereas knockdown of Axl and Mer tyrosine kinase (Mertk), which are members of the Tyro3-Axl-Mer (TAM) receptor kinase family, was found to impair phagocytosis, which was not consistent with the results in the studies described in the previous paragraph. It is unclear whether the different results on the involvement of MEGF10 in phagocytosis are due to different types of cells being phagocytosed or by different experimental methods. However, it is possible that astrocytes identify cell debris via several receptors and that the degree of involvement of each receptor varies depending on the target of phagocytosis.

Mertk is also expressed in microglia and Mertk deficiency delays the timing of the initial contact of microglia with apoptotic neurons [3]. However, with or without Mertk, apoptotic neurons were eventually removed, suggesting that microglial Mertk plays an important role in the speed of recognition or ingestion of cell debris. Moreover, the precise role of astrocytic Mertk in phagocytosis remains to be clarified.

As described above, astrocytes phagocytose cell debris via BAI1, MEGF10 and Mertk, whereas microglia phagocytosis is mediated by various receptors in addition to Mertk. Receptors that are differentially expressed between microglia and astrocytes, such as MEGF10, may be responsible for the differences in phagocytic patterns between microglia and astrocytes. For example, Damisah et al. argued that “eat me” signals presented by apoptotic soma or distal dendrites are distinct from each other and may be recognized by receptors specific to microglia or astrocytes, respectively [3]. With regard to astrocyte phagocytosis, it has been reported that the soma and neurites of a single neuron are separately identified and phagocytosed by astrocytes, probably via different receptors [11]. In the larval brain, Draper is required for the phagocytosis of vCrz^+^ neuronal soma, whereas Draper is not required for the phagocytosis of vCrz^+^ neurites [11].

These findings suggest that a variety of receptors are essential for the accurate regulation of phagocytosis. Although the types of astrocyte receptors involved in phagocytosis have been reported to be fewer than those of microglia at present, it is possible that many more astrocyte receptors are actually involved in phagocytosis. For example, a study examined the phagocytic ability of astrocytes by transcriptome analysis and reported that not only Mertk and MEGF10 but also Fc receptors were abundantly expressed in astrocytes, suggesting that Fc receptors may also regulate astrocyte phagocytosis [1]. In addition, scavenger receptors have also been found to be expressed in astrocytes and may be involved in phagocytosis [12].

Shear stress is a factor other than receptors that controls astrocytic phagocytosis. In primary astrocyte cultures, when UV irradiation induces apoptosis in a single astrocyte, the migration and phagocytosis of neighboring astrocytes were observed [13]. The results showed that almost all astrocytes within 20 μm of the irradiated point phagocytosed apoptotic cells, and 40% of astrocytes that were 40 μm away from the irradiated point phagocytosed apoptotic cells. In contrast, in the presence of shear stress caused by the flow of the medium, all astrocytes phagocytosed apoptotic cells even if they were 40 μm away from the irradiated point. Moreover, shear stress shortened the latency of phagocytosis after apoptosis induction. Astrocytes may detect shear stress through mechanosensors such as integrins and cadherins [14,15]. These findings indicate that phagocytosis is regulated not only by chemical signals through receptors but also by nonchemical signals.

### 2.2. Degradation

In general, ingested proteins are degraded through enzymatic reactions. Even during the process of degradation, the pattern of degradation is different between microglia and astrocytes. For cell debris, the time required for degradation by microglia is shorter than that required for degradation by astrocytes. In vivo two-photon imaging revealed that a single apoptotic neuron is eliminated by microglia approximately 20 h after the induction of apoptosis, whereas the same cell is eliminated by astrocytes more than 50 h later [3]. Consistent with this observation, when cell debris was added to the culture system and the disappearance of aggregated nuclei was used as an indicator of cell debris removal, primary cultured splenic macrophages removed cell debris in 3 days, whereas primary cultured astrocytes took 12 days to remove cell debris [9,16]. Lysosomal pH changes were examined by treating neuronal debris labeled with a pH-sensitive dye that fluoresces under acidic conditions. In cultured macrophages, fluorescence, which indicates transport of debris into acidic vesicles, was observed 5 h after cell debris treatment [9]. In contrast, fluorescence was hardly observed in cultured astrocytes for 12 days [16]. These results raised the possibility that the slow degradation by astrocytes was due to higher lysosomal pH. Therefore, it was examined whether degradation in astrocytes could be accelerated by treatment with PLGA Resomere RG 502H, an acidic particle that can induce acidification in lysosomes. The results showed that the degradation of cell debris by astrocytes was enhanced on the fifth day after treatment, suggesting that the high lysosomal pH of astrocytes contributes to their slow degradation. Based on this result, Lööv et al. proposed that astrocytes suppress degradation by maintaining high lysosomal pH to present antigens. Although this hypothesis is very interesting, there have been no studies to date that report antigen presentation by astrocytes, and it is necessary to clarify whether microglia or astrocytes present antigens and whether the pH of lysosomes is associated with antigen presentation.

In pathological models, a tendency for astrocyte-mediated phagocytosis to take longer than microglia-mediated phagocytosis has been reported. In the ischemic brain induced by middle cerebral artery occlusion (MCAO), microglia and astrocytes phagocytose cell debris mainly in two areas: the core region, where severe ischemia occurs, and the penumbra region, where cells survive immediately after infarction despite being exposed to an increased risk of infarction [17]. The temporal changes in the expression levels of phagocytic markers, such as galectin-3, or lysosomal markers were investigated. As a result, phagocytosis markers in microglia peaked on the third day after MCAO and returned to control levels after 14 days, while in astrocytes, these markers peaked on the seventh day after MCAO and remained high even after 14 days. It remained unclear whether lysosomal pH was involved in the temporal differences in the expression of phagocytic markers in this study. Overall, it has been suggested that microglia degrade cellular debris more rapidly than astrocytes, regardless of physiological or pathological conditions. The difference in the rate of degradation between microglia and astrocytes may be due in part to the different ways in which each cell exerts its cellular functions. Microglia are highly plastic capable of polarizing their responses such as chemotaxis and phagocytosis at detriment to other cellular processes and gene expression changes. Astrocytes on the other hand are massively multitasking cells, capable of performing numerous functions and may also be polarized into a reactive spectrum under certain conditions [18]. It would be interesting if such differences in the way microglia and astrocytes function were due to differences in their cellular origin, i.e., whether they are derived from mesoderm or ectoderm. If we can discover the mechanism that controls the difference in the way microglia and astrocytes function and control it, i.e., if we can give microglia the ability to multitask, we may have a new way to control brain functions.

Since lysosomal pH is regulated by the Na^+^/H^+^ exchanger and V-ATPase expressed in the lysosomal membrane, different types and expression levels of lysosomal membrane proteins in microglia and astrocytes may result in different lysosomal pH levels [19]. Although microglial or astrocytic molecules that are important for the regulation of lysosomal pH have been reported, it is not clear how their expression patterns are regulated in each cell type [20,21]. Therefore, to understand the different patterns in phagocytosis-induced degradation in microglia and astrocytes, it will be necessary to comprehensively identify lysosomal membrane proteins and directly compare them between microglia and astrocytes. However, we are currently lacking detailed knowledge. For example, despite the frequent use of CD68 as a lysosomal marker, its function is often not fully understood [22]. Furthermore, the study of lysosomes is profound because lysosomes exhibit heterogeneous morphology, distribution, and function even within a single cell [23]. In the future, it will be necessary to clarify in detail the characteristics of lysosomes in glial cells, including the membrane proteins and enzymes that make up lysosomes.

### 2.3. The Fate of Both Phagocytes and Phagocytosed Targets after Ingestion

Some studies have reported that soluble factors released from microglia and astrocytes in vitro are altered after the ingestion of cell debris; in a study by Magnus et al., lipopolysaccharide (LPS) stimulation was performed, and the production of tumor necrosis factor-α (TNF-α), which is an inflammatory cytokine, was compared between microglia and astrocytes [24]. The results showed that the production of TNF-α was lower when cultured microglia were treated with apoptotic thymocytes than when nonapoptotic thymocytes were added. On the other hand, thymocytes were also taken up by cultured astrocytes, but there was no difference in the amount of TNF-α produced when apoptotic or nonapoptotic thymocytes were added. Similarly, the expression level of major histocompatibility complex 2 (MHC2), a machinery protein for antigen presentation, was higher in microglia treated with apoptotic thymocytes than in those treated with nonapoptotic thymocytes, whereas there was no change in the astrocyte cultures [4]. These results suggest that microglia may be more sensitive to apoptotic cell debris than astrocytes.

Although they are not microglia, cultured blood monocyte-derived macrophages release Fas ligand, a member of the TNF family that induces apoptosis, in response to processing apoptotic neutrophils [25]. Furthermore, when supernatant containing Fas ligand is applied to neutrophils, neutrophils undergo apoptosis. Thus, macrophages that phagocytose apoptotic neutrophils are able to induce cell death in living neutrophils via the release of Fas ligand. This sequence of events is thought to be a negative feedback system in which macrophages eliminate activated neutrophils, thereby contributing to the cessation of inflammation. These findings indicate that microglia and macrophages that phagocytose apoptotic cell debris receive a signal from apoptotic cell debris and respond by reducing inflammation.

Where do phagocytosed targets go after they are degraded in lysosomes? Recent studies have shown that degradation products activate intracellular signaling, followed by the induction of an anti-inflammatory response and translocation of the degradation products. Studying the mechanism of remyelination with a focus on sterol synthesis, Berghoff et al. reported that microglia phagocytose cholesterol-rich myelin fragments, resulting in the inhibition of cholesterol synthesis in microglia [26]. As a result, the synthesis of desmosterol, a cholesterol precursor, is enhanced, and the increased desmosterol activates liver X receptor (LXR) signaling, which ultimately limits inflammation and promotes cholesterol efflux. These results suggest that phagocytosed targets can cause functional changes in phagocytes. As mentioned previously, microglia tend to phagocytose apoptotic neuronal soma, while astrocytes tend to phagocytose distal dendrites [3]. Microglia degrade DNA and cytoplasmic components at a relatively high rate, whereas astrocytes degrade lipid membranes abundantly, which may account for the different behaviors of the two cell types after phagocytosis.

## 3. Phagocytosis of Amyloid-Beta (Aβ)

Aβ is a 38–43 amino acid polypeptide derived from the processing of amyloid precursor protein (APP). Since Aβ deposition is often observed in the brains of Alzheimer’s disease (AD) patients, the Aβ clearance pathway has been presumed to be a therapeutic target for AD, and efforts have been focused on revealing Aβ clearance mechanisms [27]. Since microglia and astrocytes have phagocytic abilities, both are thought to contribute to the removal of Aβ by phagocytosis. However, the detailed cellular and molecular mechanisms by which microglia and astrocytes phagocytose Aβ and whether microglia and astrocytes have different roles in Aβ phagocytosis are largely unknown.

### 3.1. Recognition by Receptors

**Figure 2:**
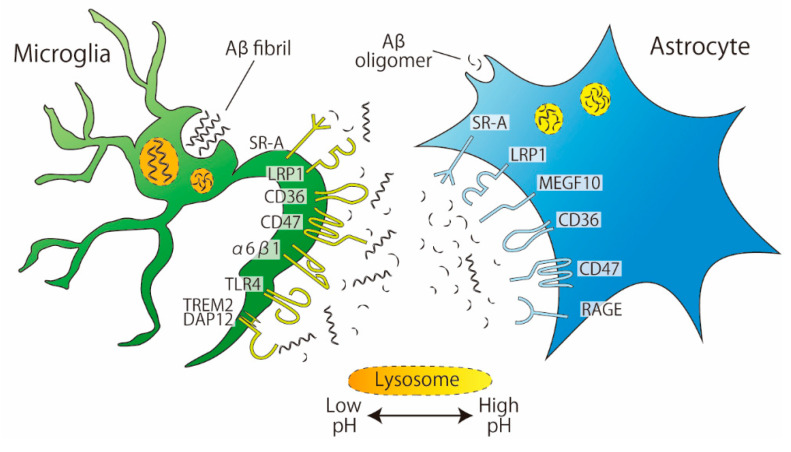
**Phagocytosis of Aβ by microglia and astrocytes.** Aβ fibrils are phagocytosed more by microglia than by astrocytes. Microglia and astrocytes share the same receptors for Aβ phagocytosis: SR-A, LRP1, CD36, and CD47. MEGF10 are expressed in astrocytes. The pH of microglial lysosomes is lower than that of astrocytes.

Studies using cultured cells from rats or mice have revealed the presence of some astrocytic receptors involved in the phagocytosis of Aβ (Figure 2). These receptors include scavenger receptor-A (SR-A, a trimeric integral membrane glycoprotein that binds directly to Aβ), low-density lipoprotein receptor-related protein 1 (LRP, which is involved in several cellular processes including lipid homeostasis or endocytosis), CD36 (a glycoprotein that acts as a receptor for various ligands), CD47 (an integrin-associated protein that has a role in cell adhesion), and receptor for advanced glycation end products (RAGE, a member of the immunoglobulin superfamily that mediates inflammation) [28,29,30]. SR-A is also expressed in microglia but is engaged in Aβ uptake in a slightly different manner in microglia than in astrocytes. The SR-A antagonist fucoidan inhibited the phagocytosis of monomeric Aβ_1–42_ by cultured rat astrocytes but not by primary cultured murine microglia [30]. In contrast, in both cell cultures, the phagocytosis of oligomeric Aβ_1–42_ was inhibited by fucoidan, indicating that the involvement of SR-A is complicated by the degree of Aβ aggregation. In addition to SR-A, microglia have a number of receptors that recognize Aβ [31]; many of the receptors expressed on astrocytes, such as LRP1, CD36, CD47, and RAGE, are also expressed on microglia. To date, the astrocyte-specific receptor for Aβ is MEGF10, which appears to preferentially mediate the uptake of Aβ_42/43_ but not Aβ_40_ [32]. Recently, among Aβ-associated microglial receptors, triggering receptor expressed on myeloid cells 2 (Trem2) has got attention as a risk factor for several neurodegenerative diseases, including AD. Trem2 enhances microglial phagocytic capacity and anti-inflammatory cytokine expression [33]. Furthermore, Trem2 induces Aβ plaque-associated microglia, which are referred to as disease-associated microglia (DAM), suggesting that Trem2 has an essential role in AD [34].

In addition to receptors, soluble factors also affect the phagocytosis of Aβ. Mulder et al. compared the phagocytosis of Aβ fibrils and oligomers using human-derived cultured microglia and astrocytes [35]. The results showed that microglia and astrocytes phagocytosed Aβ oligomers to the same extent, whereas Aβ fibrils were phagocytosed more by microglia than by astrocytes. Next, apolipoprotein E (ApoE), which acts as a ligand for LRP1, was administered with Aβ oligomers or fibrils in culture. As a result, ApoE decreased the amount of phagocytosis of Aβ fibrils by microglia but did not change the amount of phagocytosis of Aβ oligomers. In contrast, in astrocyte cultures, ApoE decreased the phagocytosis of Aβ oligomers but not Aβ fibrils. LRP1, the receptor for ApoE, is abundantly expressed on both microglia and astrocytes [36]. The difference in ApoE-induced Aβ phagocytosis between microglia and astrocytes cannot therefore be explained by the presence or absence of LRP1. In addition to the expression pattern of LRP1 in each cell type, the involvement of factors other than the receptor will need to be examined in future studies.

These in vitro studies have provided valuable insights into the differences in Aβ phagocytosis due to differences in Aβ structure. Furthermore, examination in vivo may also be required; in a recent study by Prakash et al., Aβ labeled with a pH-sensitive dye that fluoresces under acidic conditions was observed to be phagocytosed by both microglia and astrocytes in vivo [37]. The results showed an increase in fluorescence in the cell bodies of both microglia and astrocytes, indicating Aβ phagocytosis, within 20 min after the application of Aβ. To further evaluate the amount of Aβ phagocytosed by each cell, the authors measured the colocalization of fluorescent Aβ with ionized calcium-binding adapter molecule 1 (Iba1) or glial fibrillary acidic protein (GFAP), which are markers of microglia or astrocytes, respectively, at 1.5 and 3 h after Aβ was injected into the brain. The results showed that microglia phagocytosed Aβ more prominently than astrocytes. Note that the staining image showing GFAP, a filament protein, did not show the entire astrocyte structure and that the pH-sensitive dye phagocytosed by astrocytes may not fluoresce due to insufficient acidification of astrocytic lysosomes, which may underestimate Aβ uptake by astrocytes. Nevertheless, in vivo comparison of Aβ phagocytosis by microglia and astrocytes has the potential to provide important information, such as the significance of role sharing or phagocytosis, which could be observed only in vivo, as in the study by Damisah et al. Since the dynamics of Aβ in vivo are expected to be complex, it may be difficult to predict Aβ phagocytosis in vivo based solely on findings obtained in vitro. In the future, we should try to control Aβ phagocytosis by cell type-specific manipulation in vivo, making full use of the knowledge of the cellular and molecular mechanisms underlying the phagocytosis of Aβ in vitro.

### 3.2. Degradation

Transcription factor EB (TFEB), a master regulator of lysosome biogenesis, has been studied in both microglia and astrocytes with respect to the molecular mechanisms that regulate the function of lysosomes, including lysosomal membrane proteins or enzymes. TFEB binds to a sequence called the coordinated lysosomal expression and regulation (CLEAR) motif, which many lysosome-related genes have in their promoter regions, and subsequently promotes the transcription of many downstream lysosome-related genes [38]. In vitro, overexpression of TFEB in astrocytes has been shown to increase the expression of lysosome-related enzymes and promote Aβ degradation, and in vivo overexpression of TFEB in astrocytes has been shown to reduce Aβ plaques [39]. Similarly, lentivirus-mediated overexpression of TFEB in primary cultured microglia promoted the degradation of fibrillar Aβ, while knockdown of TFEB by siRNA transfection suppressed the degradation of fibrillar Aβ [40]. As a further regulator of TFEB in microglia, progranulin (PGRN, a precursor of granulin that is involved in various biological reactions, such as phagocytosis and inflammation) has been reported to suppress TFEB and inhibit lysosome biogenesis. Knockout of PGRN promotes the upregulation of lysosome-related genes such as lysosomal-associated membrane protein 1 (Lamp1) induced by traumatic brain injury [41]. In addition, the expression patterns of cathepsins, a group of acidic proteases in lysosomes, vary due to PGRN KO; in 3-month-old mice, microglia-specific expression of cathepsin D (CatD) was decreased and precursors of cathepsin L (CatL) and cathepsin B (CatB) were increased in PGRN-KO mice, suggesting that cathepsin-mediated degradation was impaired in microglia [42]. In contrast, at 12 months of age, CatD expression in microglia was unchanged, but CatD function and expression were increased in cells other than microglia. This result may be a compensatory response by other brain cells, including astrocytes, to defective degradation in microglia. Although PGRN is also expressed in astrocytes, it is more abundant in microglia, and the effect of astrocytic PGRN on TFEB and phagocytosis is not clear [43]. Additionally, in microglia and astrocytes, TFEB is commonly activated by Sirtuin 1 (SIRT1, a histone deacetylase)-mediated deacetylation, resulting in enhanced phagocytosis [40,44]. Although TFEB regulates the transcription of all lysosome-associated proteins in both microglia and astrocytes, the slight differences in its regulation by the upstream PGRN and downstream CLEAR networks may lead to differences in the lysosomal degradation capacities of microglia and astrocytes.

*Ctsb*, *Ctsd,* and *Ctsl*, encoding CatB, CatD, and CatL respectively, are AD risk genes. Single-nucleus RNA sequence of 5xFAD transgenic mice, which recapitulate major features of AD amyloid pathology, clarified that these genes are included in both DAM and disease-associated astrocytes (DAA) signature genes [2]. These results highlight the contribution of both microglial and astrocytic degradation capacities to AD pathology.

In addition to TFEB, cAMP and Zn^2+^ have been reported to regulate lysosomal functions in astrocytes [45]. In cortical astrocyte cultures, treatment with bafilomycin A1 (BafA1), a v-ATPase inhibitor, increases the pH of astrocyte lysosomes, but treatment with cilostazol (a phosphodiesterase inhibitor that inhibits the degradation of cAMP) or cAMP treatment reacidifies lysosomes. Lysosomal reacidification was inhibited by treatment with TPEN, a Zn^2+^ chelator. Simultaneous treatment with Aβ and cilostazol decreased Aβ accumulation in astrocytes compared to treatment with Aβ alone, suggesting enhanced degradation. The mechanism by which cAMP and Zn regulate the pH of lysosomes is not clear, and it is also unclear whether the acidity of microglial lysosomes is controlled by a similar mechanism.

### 3.3. The Fate of Both Phagocytes and Phagocytosed Targets after Ingestion

In both microglia and astrocytes, Aβ phagocytosis induces a variety of changes, including the release of cytokines and the production of ROS [31]. Here, we focus on the effects of Aβ phagocytosis on the phagocytic capacity of each cell and the subsequent fate of Aβ.

In primary cultured rat microglia, Aβ treatment reduced nuclear TFEB and suppressed lysosomal acidification, while total TFEB expression levels were unchanged [46]. This result was consistent with the observation that TFEB transcription in microglia was impaired within 6–12 months after birth, when Aβ plaques began to accumulate in APP/PS mice, and with the observation that nuclear TFEB decreased with the progression of Braak stage, a classification of AD stages based on the brain distribution of neurofibrils in the human brain [47,48]. Similarly, treatment of primary cultured mouse astrocytes with Aβ attenuated lysosomal acidification and CatB activation [45]. Furthermore, Aβ-containing synaptosomes from APP/PS1 mice decelerated the rate of synaptosome uptake and synaptic protein degradation compared to synaptosomes from WT or tau-accumulating mice. These effects may be attributed to the downregulation of Mertk and MEGF10 expression due to Aβ phagocytosis [49]. Overall, the phagocytic capacities of both microglia and astrocytes are probably impaired due to Aβ uptake.

What happens to Aβ that is phagocytosed by microglia and astrocytes? Microvesicles isolated from the supernatant of astrocytes and microglia cocultures treated with Aβ_42_ for 6–12 days increased the number of TUNEL (dead cell marker)-positive cells in neuronal cell cultures, suggesting the induction of neuronal death by microvesicles [50]. ELISA analysis showed that the microvesicles contained truncated forms of Aβ_42_, Aβ_1-x_, and Aβ_x-42_. These results suggest that Aβ is still toxic even after partial degradation, although there is a possibility of cytokine contamination. Similarly, tau, another protein that accumulates abnormally in AD, has been found to be propagated in the brain after being phagocytosed by microglia and released in exosomes [51]. These studies highlight that phagocytosis itself is not the ultimate goal of Aβ and tau removal. In addition to the release of exosomes and microvesicles, it is also possible that the contents of lysosomes are released directly into the extracellular space, but this has not been verified [52]. Overall, few studies have followed the dynamics of Aβ after phagocytosis, and important questions remain as to why Aβ is released without being completely degraded or how the dynamics of Aβ after degradation change with aging. Additionally, the difference between microglia and astrocytes in this process is also unknown. Therefore, clarification of the fate of Aβ after phagocytosis and the involvement of both cell types may lead to novel therapeutic strategies for AD.

There are interesting findings regarding the phagocytosis of cellular debris in AD. Electron microscopy has shown that in the brains of APP/PS1 mice, astrocytes phagocytose dystrophic neurites, but microglia rarely phagocytose these structures [53]. It has also been reported that microglia do not phagocytose Aβ plaques but accumulate around them to form a barrier [54]. In conclusion, many mysteries remain as to what and how glial cells selectively phagocytose in the context of AD. It should be noted, however, that depending on the timing of the observation, the possibility of phagocytosis by microglia remains.

Recent findings have also shown that intraperitoneal injection of interleukin-33 (IL-33) into APP/PS mice induces some microglia to become highly motile [55]. After IL-33 injection, IL-33-responsive microglia, which highly express MHC2, approached Aβ plaques and phagocytosed Aβ. Although it is not clear how MHC2 affects Aβ phagocytosis, the finding that MHC2 KO exacerbates amyloid pathology in AD model mice suggests that MHC2 is important for the phagocytic removal of Aβ [56]. These findings are important in that they demonstrate the heterogeneity of microglia in the context of phagocytosis, although it should be noted that astrocytes also express MHC2 [57]. To elucidate the heterogeneity of microglia, it is important to use the latest technologies, which have undergone remarkable innovations in recent years, such as large-scale transcriptional analysis and in vivo imaging techniques.

## 4. Phagocytosis of Synapses

The number of synapses is regulated by synaptic pruning not only during adulthood but also during development, and many studies have shown that synaptic phagocytosis by microglia plays a key role in synaptic pruning [58,59]. However, the exact role and significance of microglia and astrocytes in synaptic phagocytosis remain to be clarified. Here, we focus on the dorsal lateral geniculate nucleus (dLGN) and the hippocampus, where synaptic phagocytosis by both microglia and astrocytes has been reported.

### 4.1. The dLGN

In the developing dLGN, excess synapses formed by axonal terminals projecting from retinal ganglion cells (RGCs) undergo synaptic pruning; in P6, there are excess projections from RGCs in the left and right eyes, but axonal projections from the left and right eyes eventually separate, and eye segregation occurs due to the phagocytosis of axon terminals by microglia. The method of injecting fluorescent dye-labeled cholera toxin subunit B (CTB) into the eye as an axonal tracer and examining the phagocytosis of axonal terminals by glial cells in the dLGN is often used. The involvement of the classical complement pathway has been suggested because eye segregation is incomplete in the dLGN of C1q-KO mice [60]. Furthermore, a study focusing on the expression of complement receptor 3 (CR3), a receptor for C3, which is a downstream molecule of C1q in the classical complement pathway, in microglia revealed that microglia perform synaptic phagocytosis in the dLGN via CR3 [59]. Furthermore, KO of C4, another complement pathway component, reduced the amount of microglial phagocytosis of axon terminals in the dLGN, supporting the importance of microglia and the complement pathway in eye segregation [61]. Signals activated by CD47 and signal regulatory protein α (SIRPα), which are expressed at presynapses and in microglia, respectively, act as “do not eat me” signals that inhibit synaptic phagocytosis by microglia [62]. Furthermore, sushi repeat protein X-linked 2 (SRPX2) is expressed in neurons and binds directly to C1q to inhibit synaptic pruning via the complement pathway [63]. Thus, synaptic phagocytosis by microglia is regulated by a variety of molecules, mainly those of the complement pathway.

Some of these phagocytosis-related proteins may be involved in the selective phagocytosis of presynapses and postsynapses. CR3 KO changed the amount of vGlut2 (RGC axonal terminal-specific presynaptic marker in the dLGN) but not GluR1 (postsynaptic marker), indicating that presynapses are preferentially phagocytosed via microglial CR3, while postsynapses are not [59]. Furthermore, CD47 may selectively regulate microglial phagocytosis of vGlut2-containing synapses, as KO of CD47 reduced the density of synapses composed of vGlut2 and another postsynaptic marker, Homer1, but did not change the density of synapses composed of vGlut1 and Homer1 [62].

In astrocytes, KO of MERTK and MEGF10 reduced phagocytosis, suggesting that these receptors are required for the phagocytosis of synapses by astrocytes [64]. MERTK interacts with ligands such as proteinS, GAS6, and galectin3, but it is not clear how these ligands are involved in synaptic phagocytosis. MEGF10 interacts with C1q when astrocytes phagocytose dead cells, but C1q KO did not change the number of synapses phagocytosed by astrocytes, suggesting that C1q does not act as a ligand during the phagocytosis of synapses by astrocytes [8]. ApoE has been investigated as another protein that regulates synapse–receptor interactions [65]. There are three isoforms of ApoE: ApoE2, ApoE3 and ApoE4. ApoE2 KI increased the amount of astrocyte phagocytosis of RGC axon terminals in the dLGN, while ApoE4 KI decreased it. Thus, these findings suggest that different ApoE isoforms affect synaptic phagocytosis by astrocytes differently. Furthermore, the effect of each ApoE isoform on astrocyte uptake capacity was examined using synaptosomes and primary cultured astrocytes from APOE-KO mice. Astrocyte-conditioned medium (ACM) from ApoE2-KI mice, which contain ApoE2, significantly enhanced the binding of astrocytes and synaptosomes compared to that of ACM from ApoE3- or ApoE4-KI mice. This alteration was observed 1 h after treatment; hence, ApoE is more likely to regulate phagocytosis by affecting the interaction between synapses and astrocytes than gene expression in astrocytes. Although astrocytes have several ApoE receptors, including LRP1, only treatment with recombinant ApoE2, 3, and 4 failed to exhibit isoform-dependent modulation of phagocytic capacity, suggesting that these receptors were not involved in synaptic phagocytosis [28]. Rather, the modulation of phagocytic capacity by recombinant ApoE required protein S. Considering these data, ApoE and protein S might cooperatively contribute to synaptic phagocytosis by astrocytes.

Currently, it is thought that microglia phagocytose RGC synapses formed in the dLGN via the complement pathway, CD47, and SRPX2, while astrocytes phagocytose RGC synapses via proteins other than those of the complement pathway, such as MERTK and MEGF10. Thus, if the population of synapses with marker molecules required for synaptic phagocytosis by microglia is different from the population of synapses with such molecules for astrocytes, then microglia and astrocytes can phagocytose the specific synapses in the dLGN. On the other hand, if all RGC synapses in the dLGN have molecules that are recognized by both microglia and astrocytes, then it is possible that synapses are randomly phagocytosed by either cell. To answer these questions, it is important to investigate the mechanisms of molecular expression at the synapses being phagocytosed. For example, the report that C1q colocalizes with synapses expressing caspase-3 is very interesting from the perspective of synaptic apoptosis [66]. In addition, more detailed studies on the properties of phagocytosis-related proteins on glia are needed. For example, what astrocytic MERTK and MEGF10 recognize during synaptic phagocytosis is also an open question.

Neural activity is also an important regulator of synaptic phagocytosis by glia. Pharmacological manipulation of the strength of the input from RGCs in one eye makes the axon terminal with weaker RGC-derived input in the other eye more susceptible to phagocytosis by both microglia and astrocytes [59,64]. At present, it is not clear whether there are any differences in the neural activity sensed by these two cell types, such as the frequency or duration of neural activity. Recent findings suggest that spines with locally increased Ca^2+^ tend to disappear in a mouse model of multiple sclerosis and that their removal is mediated by microglia [67], but the more detailed mechanisms downstream of Ca^2+^ and whether astrocytic phagocytosis is also induced are not clear.

### 4.2. The Hippocampus

In the hippocampus, the phagocytosis of synapses by microglia has been reported both during development and adulthood. KO of CX3C chemokine receptor 1 (CX3CR1), which is highly expressed in macrophages, including microglia, increased CA1 pyramidal cell spines and postsynaptic density protein 95 (PSD95, a postsynaptic marker) at postnatal day 15 (P15), suggesting CX3CR1-mediated phagocytosis of synapses [58]. Furthermore, KO of TREM2, which is exclusively expressed in microglia in the brain parenchyma, increased the vGlut1 and PSD95 signals in CA1 at P18–20 and decreased the PSD95 signals encapsulated in CD68, suggesting that TREM2 is also involved in the phagocytosis of synapses [68]. In contrast, in the adult stage, astrocytes but not microglia are reported to be responsible for the phagocytosis of synapses in the hippocampus via MEGF10. Lee et al. performed tamoxifen-induced MEGF10 KO in 8-week-old mice and found that the number of vGlut1^+^ and PSD95^+^ synapses in the CA1 region increased 4 weeks after tamoxifen injection [69]. On the other hand, removal of microglia by the CSF1 receptor inhibitor PLX3397 from 8 to 12 weeks of age did not change the number of synapses, which was consistent with a previous report [70]. Furthermore, it has been reported that the uptake of synapses by astrocytes is greatly increased compared to that by microglia in TREM2-KO mice at 1 month of age [71], suggesting that astrocytes compensate for insufficient microglial-mediated synaptic phagocytosis.

Overall, at least for hippocampal synaptic phagocytosis, it appears that microglia are predominantly responsible during development, when the number of synapses changes dynamically, and astrocytes play a leading role during adulthood, when the number of synapses is maintained constant. When CX3CR1 and Trem2 are deficient, no aberrant synapses are observed in adulthood, despite an increase in synapses during development. This is thought to be due to astrocyte-mediated regulation of synaptic density to normal levels [58,68,71].

It is reasonable to hypothesize that astrocytes predominantly phagocytose synapses during adulthood in terms of spatial relationship of microglia and astrocytes with synapses. Astrocytes constantly contact pre- and postsynapses with fine processes, forming tripartite synapse, and bidirectionally communicate with synapses [72]. The close proximity may facilitate the turnover of synapses, not synapse loss, via astrocytic phagocytosis, resulting in maintaining homeostasis of synapses. During development in dLGN, astrocytes do not directly phagocytose synapses but support synaptic phagocytosis by microglia via TGF-β signaling [73]. Given that the ultrastructure of tripartite synapses probably emerge after postnatal 3 weeks, when astrocytes and synapses show mature morphology, it makes some sense that astrocytic phagocytosis is rarely observed during developmental periods [74]. It should be noted though that synaptic phagocytosis by microglia during adulthood occur in some specific contexts. A study using a contextual fear conditioning paradigm in adult mice showed that depletion of microglia after a training session increased freezing behavior in a test session, indicating that forgetting of fear memory is prevented [75]. In addition, microglial depletion increased the synapses on engram cells, but the inhibition of engram cell activity or the complement pathway abolished the increase. These results suggest that microglia regulate forgetting in an activity- and complement-dependent manner in adults through synaptic phagocytosis.

## 5. Conclusions

In this review, we compared the phagocytic functions of microglia and astrocytes, which have attracted much attention in recent years, from the viewpoint of their different roles in phagocytosis. As a result, we found that there were detailed differences in the mode of phagocytosis and related proteins between these two cell types. More specifically, astrocytes and microglia may perform different homeostatic functions in terms of regulating neural networks and their activities. For example, astrocytes may be biased toward the maintenance and efficiency of synaptic transmission, whereas microglia play a more active role in regulating brain functions such as learning and memory by controlling the removal and formation of synapses. Whether these differences are because microglia and astrocytes are derived from the mesoderm and ectoderm, respectively, and have different intrinsic cellular characteristics, was not determined but will be interesting to study in the future.

Microglia are highly motile cells that express a wide variety of receptors for the rapid detection of abnormalities such as cell death and pathogen invasion in the brain. Microglia are also capable of phagocytosing relatively large structures because they can retract their protrusions and adopt ameboid shapes. On the other hand, astrocytes, which are important in maintaining the physical structure of the brain and have a role in supporting the function of adjacent neurons, especially neurotransmission, [76] are thought to exert their phagocytic function without significantly altering their cellular structure. Taken together, these findings suggest that microglia and astrocytes perform phagocytosis in a manner appropriate for each cell’s original function and morphology. Furthermore, it is becoming clear that there is crosstalk between microglia and astrocytes to regulate phagocytosis [71,77]. It is clear that microglia and astrocytes play cooperative roles in phagocytosis; however, how and in what manner these roles are shared remains to be elucidated. As microglia- and astrocyte-specific genetic manipulations become easier and more reliable, it will be possible to directly compare the functions of these two cell types in phagocytosis based on molecular mechanisms.

Phagocytosis is undoubtedly important for the removal of unwanted proteins in the brain. However, simply promoting phagocytosis would not always be a good strategy. For example, microglia-specific KO of TAR DNA-binding protein of 43 kDa (TDP-43) enhanced the phagocytosis of amyloid plaques, but it also increased the phagocytosis of synapses [78]. Furthermore, even if the phagocytosis and degradation of Aβ by microglia is enhanced, Aβ phagocytosis by microglia will not be a therapeutic strategy for AD if the degradation products of Aβ remain toxic and are propagated by microglia in the brain. Previous studies on phagocytosis have revealed how one or more molecules are involved in the uptake of unwanted substances by microglia or astrocytes. However, further research using techniques such as high-resolution live imaging in vitro and in vivo is needed to gain a complete understanding of phagocytosis, including the uptake of unwanted substances, their degradation, and the fate of these degradation products.

Research on phagocytosis in the brain is currently underway and is in full swing. In the near future, the regulation of the phagocytic functions of microglia and astrocytes may be targeted for the treatment of brain diseases.

## Data Availability

Not applicable.
